# Administrative data underestimate acute ischemic stroke events and thrombolysis treatments: Data from a multicenter validation survey in Italy

**DOI:** 10.1371/journal.pone.0193776

**Published:** 2018-03-13

**Authors:** Marzia Baldereschi, Daniela Balzi, Valeria Di Fabrizio, Lucia De Vito, Renzo Ricci, Paola D’Onofrio, Antonio Di Carlo, Maria Teresa Mechi, Francesco Bellomo, Domenico Inzitari

**Affiliations:** 1 Institute of Neuroscience, Italian National Research Council, Florence, Italy; 2 Epidemiology Unit, Florence, Italy; 3 Agenzia Regionale Sanità, Florence, Italy; 4 Emergency Medical Services, Regione Toscana, Florence, Italy; 5 Regione Toscana, Florence, Italy; 6 Careggi Hospital, Florence, Italy; 7 Regional Health System, Florence, Italy; 8 Department of Neurology, Pharmacology and Pediatrics Department (Neurofarba), University of Florence, Florence, Italy; Universita degli Studi di Napoli Federico II, ITALY

## Abstract

**Background:**

Informing health systems and monitoring hospital performances using administrative data sets, mainly hospital discharge data coded according to International-Classification-Diseases-9edition-Clinical-Modifiers (ICD9-CM), is now commonplace in several countries, but the reliability of diagnostic coding of acute ischemic stroke in the routine practice is uncertain. This study aimed at estimating accuracy of ICD9-CM codes for the identification of acute ischemic stroke and the use of thrombolysis treatment comparing hospital discharge data with medical record review in all the six hospitals of the Florence Area, Italy, through 2015.

**Methods:**

We reviewed the medical records of all the 3915 potential acute stroke events during 2015 across the six hospitals of the Florence Area, Italy. We then estimated sensitivity and Positive Predictive Value of ICD9-CM code-groups 433*1, 434*1 and thrombolysis code 99.10 against medical record review with clinical adjudication. For each false-positive case we obtained the actual diagnosis. For each false-negative case we obtained the primary and secondary ICD9-CM diagnoses.

**Results:**

The medical record review identified 1273 acute ischemic stroke events. The hospital discharge records identified 898 among those (true-positive cases),but missed 375 events (false-negative cases), and identified 104 events that were not eventually confirmed as acute ischemic events (false-positive cases). Code-group specific Positive Predictive Value was 85.7% (95%CI,74.6–93.3) for 433*1 and 89.9% (95%CI, 87.8–91.7) for 434*1 codes. Thrombolysis treatment, as identified by ICD9-CM code 99.10, was only documented in 6.0% of acute ischemic stroke events, but was 13.6% in medical record review.

**Conclusions:**

Hospital discharge data were found to be fairly specific but insensitive in the reporting of acute ischemic stroke and thrombolysis, providing misleading indications about both quantity and quality of acute ischemic stroke hospital care. Efforts to improve coding accuracy should precede the use of hospital discharge data to measure hospital performances in acute ischemic stroke care.

## Introduction

Patients with acute ischemic stroke (AIS) are often selected for epidemiological reporting, research, and for surveillance using hospital discharge data [1], mostly based on the 9^th^ Edition-Clinical Modification of the International Classification of Diseases [2]. These data are not generated for research purposes but reflect real-world practice both at the hospital and population level, and allow cross-national comparisons. Monitoring hospital performances using hospital-discharge-data is now commonplace in several countries [3], and remains the only source of information for acute stroke everywhen ad-hoc registration systems are not affordable. Stroke coding must be valid and reliable for distinguishing the major subtypes of acute stroke, which differ from one another in terms of incidence rates, risk factors, hospital care, outcomes and costs. Therefore validation studies are essential for estimating data quality [4]. Previous studies suggest that, at variance with several cardiac events, hospital discharge data with stroke codes should be reviewed, indicating that stroke remains difficult to classify reliably [5,6]. According to a recent systematic review [3], the sensitivity of ICD9-CM codes for AIS, available from only six papers, ranged from 2% to 80%, while the Positive Predictive Value (PPV), evaluated in 20 studies, ranged from 40% to 100%.

Tuscany region (Italy) is currently implementing a coordinated system of stroke care [7] that could be monitored by the means of administrative data sets. We therefore explored actual reliability of hospital discharge data produced by ICD9-CM stroke coding. We hereby report the results of the accuracy of ICD9-CM coding for AIS across the 6 hospitals covering the entire Florence Area (FA), in Tuscany.

## Materials and methods

The FA spans 3500 Km^2^ with a total population of 838,000 inhabitants [8]. The health system is entirely financed by public regional authority and acute stroke care is structured in an hub-and-spoke service configuration with 5 hospitals authorized to administer tissue plasminogen activator (t-PA) treatments (spoke hospitals) and routing patients needing more intensive services to the one also entitled to perform endovascular interventions (hub hospital).

We estimated accuracy of ICD9-CM AIS codes in terms of sensitivity and PPV against medical record review with clinical adjudication.

### Identification of potential acute stroke events

**Fig 1:**
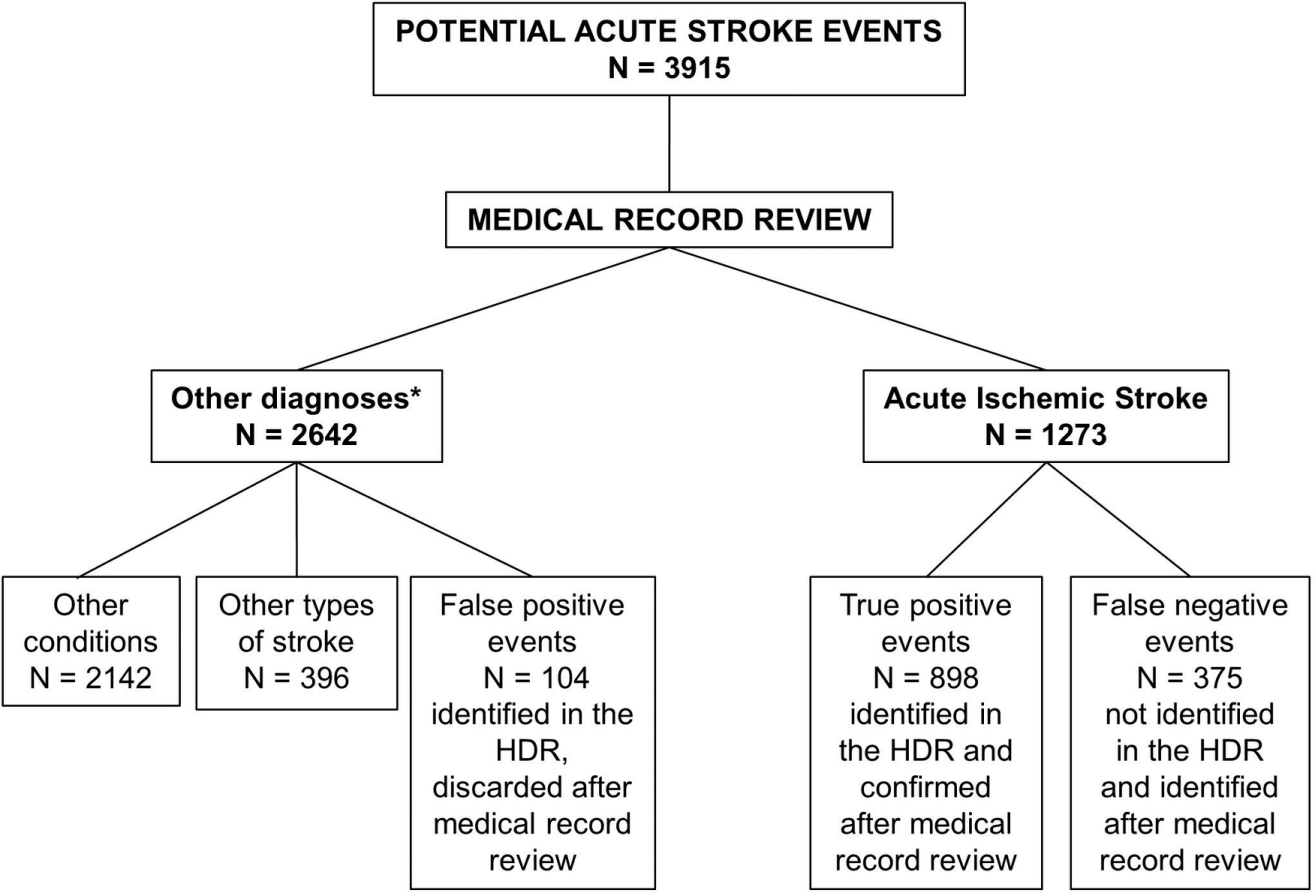
Inclusion flow-chart. *Diagnoses were: Intracerebral hemorrhage 311, Subarachnoid hemorrhage 85, Subdural hematoma 4, Other conditions 2142, including TIA, planned hospitalizations for surgery, head trauma events, epilepsy, etc. EMS: Emergency Medical Services; ED: Emergency Department; HDR: Hospital Discharge Records.

We identified all potential acute stroke (any type of stroke) events by summing up all events triaged as stroke or TIA, recorded either in the Emergency Department (ED) or in hospital discharge data with an ICD9-CM diagnostic code 430–435 (any type of stroke and TIA) in primary position, from January 1 through December 31, 2015 (Fig 1).

Selection was limited to the primary position codes to increase the likelihood of identifying acute incident rather than prevalent stroke events. For each potential stroke event an exhaustive medical record was built up (see below) and reviewed by a stroke neurologist (MB). All FA hospitals have CT scanners available in ED and could therefore provide neuroimaging to all the potential stroke events included in our study, except patients who died before reaching the hospital or the CT scanner. Repeated hospital admissions referring to the same event were dropped from selected discharges; they were defined as hospital admissions within 28 days from the previous admission or represented patients transferred from one hospital to another. We also did not include spinal and retinal arterial infarctions (ICD9-CM code 336.1 and 362.31, respectively).

### Test method: ICD-9CM codes 433*1 and 434*1 in primary diagnosis, 99.10 in any intervention position on the hospital discharge records (HDR)

In Italy, all hospitals, both public and private, are required for every episode of in-hospital care to submit a discharge diagnoses and interventions abstract, to a central administrative database. This single repository allows for accurate denominators, i.e., all hospitalizations in Italy, and is the only national source of data for stroke.

Six diagnoses and six interventions are coded by the caring physicians using [2]. The primary diagnosis is the condition that required the highest use of resources. The five secondary diagnoses are comorbidities. The discharge abstract details also patient identifier, demographics, admission and discharge dates. These abstracts are termed Hospital Discharge Records (HDR). ICD9-CM 433*1 and 434*1 are the code groups for AIS. In 1998 a new ICD9-CM intervention code, 99.10, was designated for injection or infusion of thrombolytic agents (t-PA).

We tested sensitivity and positive predictive value (PPV) of ICD9-CM codes 433*1 and 434*1 when recorded in the primary diagnosis position, as well as 99.10 code in any intervention position on the HDR, against the medical record review and adjudication (Reference method, see below).

### Reference method: Medical record review

For each potential acute stroke event (see above) an exhaustive medical record was built up by merging data from 6 sources: Emergency Medical Services (EMS) database, ED database, HDR, ED and hospital records, neuroimaging database, and hospital-pharmacy records. Medical records were reviewed by a single stroke neurologist (MB) and each event was assigned to one of the following according to the WHO-MONICA criteria [9]: acute ischemic stroke, subarachnoid hemorrhage, intracerebral hemorrhage, TIA, no acute stroke. The WHO-MONICA criteria [9] categorize potential cases as either definite stroke or not stroke, and provide criteria for stroke subtyping based on findings at brain imaging. Incomplete ascertainment of fatal events may lead to the diagnosis of acute ill-defined cerebrovascular event. Later on, 100 medical records were random sampled to make the stroke neurologist (MB) conducting a second adjudication, in order to provide 2 data points for intra-rater reliability. The sample was automatically generated by using Stata’s random-number generator, without replacement. Percent agreement turned out 92.2% with a Kappa statistics [10] of 0.90 (to account for chance agreement).

Advances in neuroimaging have resulted in many events that would have been labelled as TIA now being considered as minor strokes, but this is an area of ongoing controversy [11]. We took a conservative approach by not considering episodes of TIA as acute stroke. The t-PA treatments were identified both by reading clinicians’ note on ED and hospital record, as well as by the linked hospital-pharmacy record.

### Data linkage

Data linkage contributed in creating the medical record database for review, clinical adjudication and analyses, that is the reference method (see above).

A secure third party (the FA Epidemiology Center) carried out data linkage from the 6 databases: EMS database, ED database, HDR, ED and hospital records, neuroimaging database, and hospital-pharmacy records. These 6 databases were automatically linked using the fiscal code, a personal number that works as unique identifier all over Italy.

### Data analyses

Descriptive statistics were used to estimate the frequency of baseline characteristics. AIS events identified by both test and reference method were true-positive cases; AIS identified only by the reference method were false-negative cases; events identified as AIS on test method but not confirmed by reference method were false-positive cases.

ICD9-CM coding accuracy was estimated in terms of sensitivity and PPV. Sensitivity was defined as the proportion of true-positive cases identified by reference method also identified by test method. PPV was defined as the proportion of true-positive cases among those identified as AIS by the test method. PPV was estimated for each code group separately and altogether. Sensitivity and PPV were presented as percentages with their 95% confidence intervals (CI).

For each false-positive case we obtained the actual diagnosis. For each false-negative case we obtained the primary and secondary ICD9-CM diagnoses on the HDR.

Factors influencing the probability of being a true-positive (correctly coded as AIS on the HDR) versus the probability of being a false-negative were assessed by means of a multivariate stepwise logistic regression model (forward method with p<0.10 for entrance into and p>0.15 for removal from the model). Age, gender, EMS transportation, and hospital were entered as covariates; odds ratios (OR) and 95% Confidence Intervals (CI) were calculated. The goodness of fit of the model was checked through the Hosmer-Lemeshow test. A P value <0.05 was considered significant.

Statistical analyses were performed using the Stata/IC 14.1 software (Stata Corp, College Station, Texas, US).

The Regional Committee for Medical and Research Ethics gave the approval to conduct this study without patient consent (for each FA hospital).

## Results

### Study sample

The inclusion process (Fig 1) yielded a total of 3915 potential acute stroke events, whose medical records were reviewed by a stroke neurologist (MB) and that were diagnosed according to WHO-MONICA criteria [2]. Medical record review, the reference method, identified 1273 AIS events, and 173 t-PA treatments whose main characteristics are described in Table 1. Women accounted for the 55% of the study sample, the 76.6% of AIS events reached hospital by EMS, distribution by hospital of first arrival reflected hospital catchment area with Hub hospital admitting the 32.3% of AIS events during the study period. Only 3 in-hospital AIS could be identified. The ED correctly diagnosed and coded 76% of cases.

**Table 1:** Acute ischemic stroke patients characteristics (N = 1273).

Characteristic	
**Age, mean (SD) [IQR range]**	79.0 (11.7) [73–84]
**Female gender, N (%)**	700 (55)
**Arrival by Emergency Medical Service, N (%)**	975 (76.6)
**Hub Hospital, N (%)**	412 (32.3)
**Spoke Hospital 1, N (%)**	284 (22.3)
**Spoke Hospital 2, N (%)**	47 (3.7)
**Spoke Hospital 3, N (%)**	125 (9.8)
**Spoke Hospital 4, N (%)**	150 (11.8)
**Spoke Hospital 5, N (%)**	252 (19.8)
**In-Hospital Stroke, N (%)**	3 (0.2)
**t-PA Treated, N (%)**	173 (13.6)
**Correctly diagnosed in ED, N (%)**	966 (75.9)

### Validation of HDR diagnoses and procedures

Among the 1273 AIS events, 898 (70.5%) were correctly identified by HDR, but 375 (29.5%) were missed. There were 104 events identified as AIS on the HDR, but discarded after medical record review (false-positive cases). Sensitivity of the test method was, therefore, 70.5% (95%CI, 68.0–73.0) and the PPV was 89.9% (95%CI, 87.6–91.4). Codes 434*1were used much more often (93.7%) than 433*1 codes. Code group-specific PPV was 85.7 (95%CI, 74.6–93.3) for 433*1 and 89.9% (95%CI, 87.8–91.7) for 434*1 (Table 2).

**Table 2:** Performances of HDR to identify acute ischemic stroke events.

	Acute ischemic stroke events		HDR accuracy				
	Identified in HDR** (test method)	Identified by medical record review (reference method)	True-positive	False-negative	False-positive	Sensitivity# (95% CI)	PPV## (95%CI)
433*1	63		54		9		85.7 (74.6–93.3)
434*1	939		844		95		89.9 (87.8–91.7)
**All events** 433*1+434*1	1002	1273	898	375	104	70.5 (68.0–73.0)	89.6 (87.2–92.0)

**HDR = Hospital Discharge Records; CI = Confidence Intervals

#True-positive events/events identified by reference method

##True-positive events/events identified in HDR.

Within the medical record review, we found 97 t-PA treatments that were not coded on HDR (Table 3). Out of these 97, 30 events were not even coded as AIS in primary diagnosis position (Table 4).

**Table 3:** Performances of HDR to identify t-PA treatments.

t-PA treatments			HDR accuracy			
Identified by HDR* (test method)	Identified by ED**	Identified by medical record review (reference method)	True- positive	False- negative	Sensitivity# (95% CI)	PPV## (95%CI)
76	0	173	76	97	43.9% (38.1–51.7)	100%

*HDR = Hospital Discharge Records; CI = Confidence Intervals

**ED = Emergency Department

#True-positive events/events identified by reference method

##True-positive events/events identified in HDR.

**Table 4:** Description of the 375 discharges with acute ischemic stroke events not coded 433*1 e 434*1 in primary position (false-negative events).

Disease	ICD-9CM code	N (with t-PA treatment, n = 30)	%
No HDR*		107	28.5
**Occlusion and stenosis of precerebral/cerebral arteries WITHOUT mention of cerebral infarction**	433*0–434*0	72 (8)	19.2
**Acute but ill-defined cerebrovascular disease**	436	52 (5)	13.9
**TIA**	435	35 (5)	9.3
Acute ischemic stroke in secondary positions**	433*1–434*1	42 (4)	11.2
**Intracerebral hemorrhage**	431	7 (4)	1.9
**Other: hemiplegia, epilepsy, neoplasms, intracranial injury**	342, 345, 140–239, 850–854	60 (4)	16

*died in ED n = 3, refused hospitalization n = 8, transferred to other hospitals or long-term facilities n = 96.

**primary position diagnoses were as it follows: pneumonia n = 11, septicemia n = 4, neurological deficits n = 7, cancer(mixed) n = 5, myocardial infarction n = 6, atrial fibrillation n = 4, 2 cardiac arrest n = 2, and heart failure n = 3

None of these events were given ICD9-CM codes for other conditions that might have warranted thrombolysis (i.e., pulmonary embolus). The 4 t-PA treatments for stroke events coded as intracerebral hemorrhage (431 in primary position) did have a symptomatic intracranial hemorrhage, nevertheless AIS was not recorded in any position. The remaining 26 false-negative treated events were mainly miscoded as TIA, acute but ill-defined cerebrovascular disease (ICD9-CM code 436), occlusion of precerebral/cerebral arteries without mention of cerebral infarction (ICD9-CM codes 433*0–434*0), and hemiplegia, or coded as AIS in secondary positions. We took a careful look into the 375 false-negative events (Table 4): 107 events, lacking HDR accounted for the majority of false-negatives (28.5%), the ICD9-CM miscoding 433*0–434*0 accounted for another large proportion (19.2%). Other false-negative events (11.2%) resulted from misplacing the right codes in secondary diagnoses. As far as the 107 AIS events without HDR are concerned, we found that 73 cases were directly discharged from ED to long-term facilities, 8 cases refused hospitalization, and 3 died in ED. We were not able to track the remaining 23 cases, that were transferred to another hospital outside the FA. The 107 AIS events without HDR were excluded from the logistic regression analysis that pointed to available neurological consulting (i.e., Spoke hospital 5), and hospitalization in the Hub hospital as independent predictors of correct coding, in addition to male gender (Table 5).

**Table 5:** Factors associated with correct coding of acute ischemic strokes in the hospital discharge records. True-positive events (n = 898) versus false-negative events (n = 268).

	N	OR (95% CI)
**Male gender**	573	1.39 (1.05–1.86)
**Spoke Hospital 5 (Neurological consulting)**	252	1.78 (1.24–2.57)
**Hub hospital**	412	3.03 (2.11–4.33)

## Discussion

In the FA, the main limit to the current use of hospital discharge data for capturing AIS events was a high proportion of false-negative cases, that yielded a suboptimal level of sensitivity. PPV was satisfying, over 85% for each ICD-9CM code group used to record AIS.

Moreover, the use of procedure code 99.10 to identify t-PA treatments proved even more incomplete. This treatment is mainly administered in the ED departments, and only seldom coded in HDR.

The reasons for the high rate of missed cases are reported, and most of them can benefit from ad-hoc guidelines, training, as well as proper databasing. According to our findings, the main reasons for the undercount of AIS is the lack or the unavailability of HDR, suggesting that a more comprehensive databasing is needed to identify all AIS cases. Misclassification to non-acute precerebral and cerebral arteries occlusion (ICD9-CM codes 433*0 and 434*0), to TIA (435), and to acute but ill-defined cerebrovascular disease (436), as well as the misuse of the right codes in secondary positions, largely contribute to false-negative cases but might benefit from coding guidelines and training.

As far as the misuse of code 436 is concerned, we believe that, when CT is negative for recent brain lesions, as it usually is in the first 12 hours from onset [11], conservative clinicians may still feel more comfortable to classify these as acute but ill-defined strokes. In fact, despite the neuroimaging support, 52 (13.9% of false-negatives) AIS events were coded as “acute but ill-defined stroke”. The first CT scan performed in ED resulted negative for all the 52 events.

The availability of either neurological ward or neurological consulting, as it actually occurred both in the Hub hospital and in one of the Spoke hospitals, are the major predictors of correct AIS coding.

We found that medical record review could detect 94 (55.3%) more events treated with thrombolysis than by using 99.10 code on HDR. This is much higher than previously reported [12] and made the rate of t-PA treatments among AIS substantially higher than that based on ICD-9CM code 99.10 (13.6% vs. 6%, respectively). As reported also in US [13] patients treated in Drip&Ship procedures or treated and then transferred to another hospital for possible endovascular treatment are likely missed by HDR.

Moreover, in our survey 30 (17.6%) of treatments were also false-negative AIS events on HDR. Events coded with hemorrhagic stroke code do represent hemorrhagic transformation after receiving t-PA, but coding physicians should well understand the distinction between these two entities and code accordingly. Estimations of t-PA treatments as well as of hemorrhagic transformations based on HDR proved extremely inaccurate.

### Strengths

Most studies validating ICD9-CM stroke codes select patients who have records with the diagnostic codes of interest [3,4,14]. Fundamentally this approach evaluates the agreement between coders and re-abstractors of medical records. Variability in identifying patients with acute stroke using hospital discharge coding with ICD-9CM codes has been reported and confirmed [15–17].

Our study is likely to have identified the vast majority of AIS events in the FA, because we did not sample either hospitals or patients, we reviewed medical records of all events with cerebrovascular codes, not only ischemic stroke codes, both from ED, triage, and discharge diagnosis as well as HDR. In fact we identified a large number of non stroke-events (2246) due to our inclusive method of databasing, that allowed the identification of false- negative AIS events and therefore sensitivity estimates. We even reviewed 107 potential stroke events without HDR, because of transfer to hospitals outside the FA or long-term facilities, or dying in ED.

Case identification is based on extensive medical record review and not on ICD-9CM. We could identify a large number of AIS events, only one study could enroll a larger sample (2308 AIS) and estimate sensitivity [18], but across 6 years and using a stroke register as the gold standard.

Moreover, our study was not confined to one hospital, but could explore stroke coding across any ward of the six hospitals with more than 60 coding physicians, that are likely to be representative of different coding practices. This enhances the external validity of our findings.

Data linkage between the 6 databases proved successful, because the fiscal code allowed for data from multiple databases to be linked reliably, for comprehensive databasing.

Our experience suggests a realistic and rapidly implementable contribution to retrieve and reviewed stroke patients’ medical records.

### Limitations

The misclassification of AIS events might be influenced by stroke severity [19] that we could not include in data analyses because it is not coded and only seldom retrievable from medical records.

We had medical records reviewed and adjudicated by only one stroke neurologist, even though we partially amended this limitation providing intra-rater reliability.

Changes in clinical practice may influence documentation and coding: stroke and TIA, using discharge hospital data have been shown to be decreasing [20]. This is consistent in advances in stroke prevention but also in modification in clinical practice, with more patients with very mild stroke and TIA being evaluated and discharged from ED without hospital admission [19].

We did not aim to evaluate HDR accuracy to estimate incidence, given the inability to distinguish first-ever from recurrent stroke events. Moreover, our inclusion criteria likely missed most in-hospital AIS cases and could not capture residents that have been hospitalized outside the FA, as well as any stroke event that did not reach hospital.

## Conclusions

These findings have relevant implications for stroke surveillance. Tracking AIS by hospital discharge data proved fairly specific but insensitive, because of inaccurate coding. Up to now, these data may provide misleading indications both about quantity and quality of AIS hospital care. On the other hand, measuring the impact of stroke by the means of population-based and hospital-based registers is time-and cost-consuming, limited in sample size, duration of follow-up and geographical coverage, while hospital discharge data are continuously collected, cheaper and ideally covering the 100% of acute stroke care events. Therefore, improving the accuracy of coding should be further appraised, by the means of standard guidelines, ad-hoc training and auditing focusing on the relevance of precise diagnosis coding.

Efforts to improve accuracy in AIS coding should precede the use of hospital discharge data to measure hospital performances in AIS care.
